# LIBS as a novel tool for the determination of the imidization degree of polyimides

**DOI:** 10.1007/s00216-024-05163-6

**Published:** 2024-02-13

**Authors:** Birgit Achleitner, Laurie Girault, Silvia Larisegger, Michael Nelhiebel, Patrick Knaack, Andreas Limbeck

**Affiliations:** 1 TU Wien, Institute of Chemical Technologies and Analytics, Getreidemarkt 9/164, 1060 Vienna, Austria; 2 TU Wien, Institute of Applied Synthetic Chemistry, Getreidemarkt 9/163, 1060 Vienna, Austria; 3 KAI Kompetenzzentrum Automobil- und Industrieelektronik GmbH, Argentinierstraße 8, 1040 Vienna, Austria; 4 KAI Kompetenzzentrum Automobil- und Industrieelektronik GmbH, Technologiepark Villach Europastraße 8, 9524 Villach, Austria

**Keywords:** Laser-induced breakdown spectroscopy, Polymer analysis, Degree of imidization

## Abstract

Due to their outstanding chemical and physical properties, polyimides are widely used in industrial applications. The degree of imidization of polyimides significantly influences their properties, making it an important factor in tailoring the material for specific applications. Imidization refers to the process of converting a precursor poly(amic acid) by removing water, and it is essential to analyze this process in detail to tune the final structure and properties of the material. Conventional techniques for this task include Fourier transform infrared spectroscopy (FT-IR), thermogravimetric analysis (TGA), or differential scanning calorimetry (DSC), but they lack the possibility of spatially and/or depth-resolved analysis or do not enable in-line monitoring capabilities. To overcome these limitations, we propose laser-induced breakdown spectroscopy (LIBS) as a powerful tool for the monitoring of the imidization reaction. To establish a measurement method, a total of 130 in-house prepared, self-synthesized samples were thermally cured to exhibit varying imidization degrees. IR spectroscopy served as a reference technique during method development, and a novel formula for calculating the degree of imidization, based on the C_2_ and H signal trends, was introduced. The calculated imidization degrees of model thin films based on LIBS were in good accordance with the IR reference method although minor differences between the two methods were expected due to varying information depth and the size of the sampled area. Additionally, the robustness of the procedure was demonstrated by depth profiling of a stacked model polymer, spiking with commercially available additives and, ultimately, by analyzing industry-relevant polymer samples.

**Graphical Abstract:**

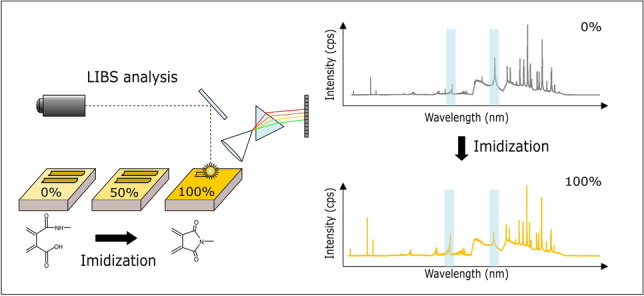

## Introduction

Polyimides are an important class of high-performance polymers characterized by an imide group as a building block of the polymer backbone. Due to their unique chemical and physical properties, they have been widely used in various industries involving high temperatures and extreme environments such as automotive, aerospace, and electronic applications. Polyimides are known for their excellent mechanical characteristics, high thermal stability, great chemical resistance against solvents and moisture, low dielectric constants, and many more. Additionally, they can be processed as films, fibers and fiber composites, engineering plastics, porous membranes, etc. and are synthesized from a huge number of different monomers. As a result, they have found extensive implementations in most high-tech fields, and the applications range from sensors, membranes, electronic displays, optoelectronic devices, batteries to anticorrosion coatings for steel and biomedical applications [1–4].

Because polyimides are generally non-soluble and will degrade at high temperatures near their glass transition temperature, their production is usually done via a two-step process. This procedure was pioneered by workers at DuPont™ in the 1950s and continues to be the primary processing route for polyimides until this day.

The first step involves the synthesis of a soluble precursor from a dianhydride and a diamine at ambient conditions in a dipolar aprotic solvent, such as N-Methyl-2-pyrrolidon (NMP) or N,N-dimethylacetamide (DMAc). This reaction yields in the corresponding poly(amic acid), which is then cyclodehydrated in a thermal or chemical process called “imidization” to form the final product [5–9]. The degree of imidization represents the percent conversion of the amic acid group into imide and is closely related to the final properties of the polymer. As gradual heating from 150 to 400°C for a certain time is necessary for complete thermal imidization and chain ordering, the quality of coatings or films depends on the production parameter such as coating method, drying temperature and rate and temperature, and imidization temperature [10–14].

Therefore, the curing of polyimide films has been studied extensively in the last decades. Popular techniques like infrared (IR) spectroscopy to analyze the reaction progress and calculate the imidization degree [10, 15, 16], dynamic scanning calorimetry (DSC) and thermogravimetric analysis (TGA) to monitor the thermal behavior during the cyclization [12, 17], and X-ray diffraction (XRD) to investigate the polymer chain orientation are applied [11–13]. These methods are well established but are either limited to bulk properties or the sample surface. IR spectroscopy in particular is challenging for colored, high-absorbing samples as well. Regarding the amount of literature on synthesis and application of polyimides, with rapid development of many high-tech industries and materials, there is an urgent need for up-to-date characterization techniques to ensure constant material properties, accompany material processing and testing, or investigate material failure.

Laser-induced breakdown spectroscopy (LIBS) has been successfully used for polymer analysis [18–21], especially within the fields of discrimination and classification [22–25] as well as polymer degradation [26, 27]. Considering that this technique can provide additional depth-resolved analysis and elemental information, it could make a valuable contribution to material testing and failure analysis of polyimides. Thus, the goal of this study was to implement a LIBS method to monitor the imidization reaction of polyimides, as well as implementing a quantitative measure to characterize material properties. A self-synthesized polyimide was used as sample material and thermally cured to obtain different conversion ratios of the amic acid vs. imide group respectively imidization degree. In order to verify our findings, IR spectroscopy served as a reference method for the determination of the imidization degree. Furthermore, we demonstrate the applicability of the developed LIBS method in the presence of additives and for commercial polyimides, as well as to study layered systems.

## Materials and methods

### Reagents

High-purity silicon wafers with a gold top layer (10 × 10 mm^2^) used as a substrate material were provided by Infineon Austria AG (Villach, Austria). Pyromellitic dianhydride (PMDA) from TCI Deutschland GmbH (Eschborn, Germany) and 4,4’-oxy-dianiline (ODA, 97%) from Sigma-Aldrich (Buchs, Switzerland) were used for synthesis of polyimide samples. N-Methyl-2-pyrrolidon (NMP, anhydrous, 99.5%) served as solvent and was obtained from Sigma-Aldrich (Buchs, Switzerland). Butylated hydroxytoluene from Merck (Darmstadt, Germany) and 2,4-dibromophenol from Honeywell Fluka (Schwerte, Germany) were used as polymer additives. Nitrogen (5.0) used during sample preparation was supplied by Messer Austria as well as Argon (5.0) used during LIBS measurements.

### Sample preparation

**Fig. 1:**
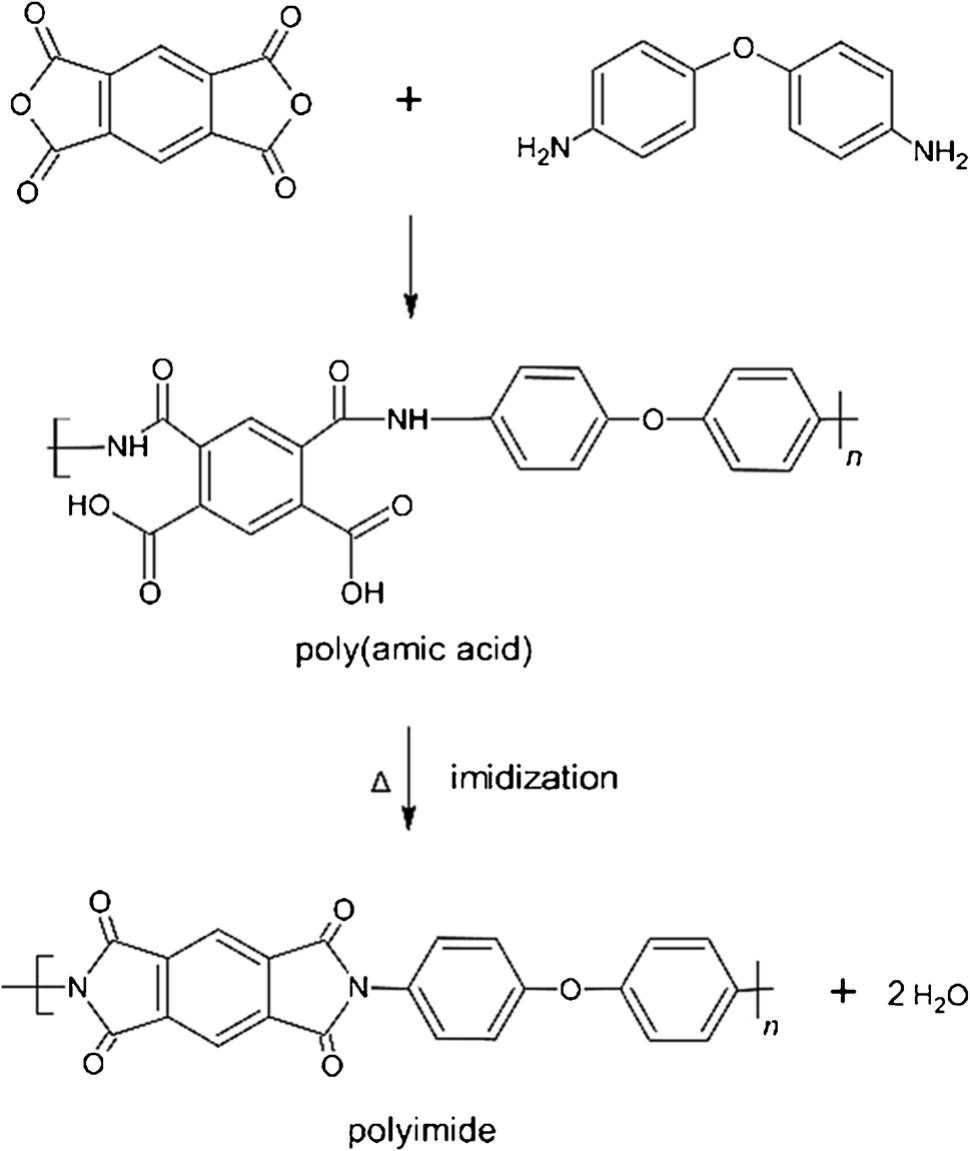
Reaction scheme for the preparation of a polyimide from pyromellitic dianhydride (PMDA) and 4,4’-oxy-dianiline (ODA)

Polyimide samples were prepared via a 2-step process, according to Fig. 1. In order to synthesize the poly(amic acid) precursor (PAA), PMDA was slowly added to an ODA/NMP solution while stirring at room temperature for 24 h under argon atmosphere. The solid content of the resulting solution was 7%.

Polymer films were prepared by casting 50 µl of the PAA solution on a gold-coated silicon wafer as substrate, whereat the substrate surface was completely covered. After solvent evaporation at 80°C in a vacuum dryer for 120 min, samples were cured under nitrogen gas flow at a specific temperature *T*_c_ ranging between 160 and 400°C for a specific time *t*_c_. In the following sections, *T*_c_ is therefore referred to as the curing temperature and *t*_c_ as the curing time. All in all, 5 different curing temperatures (160, 180, 200, 300, and 400°C) and 6 different curing times (5, 10, 15, 30, 60, and 120 min) were employed. For each combination of *T*_c_ and *t*_c_, four replicate samples were fabricated and named PI-T_c_-t_c_-1 to 4, according to the thermal treatment employed. In addition, ten samples were solely exhibited to the solvent evaporation at 80°C for 120 min and were named PAA-80–120-1 to 10. As a result, a total number of 130 specimens with a size of 10 × 10 mm^2^ was produced. The prepared polyimide films exhibited a color ranging from almost transparent to dark yellow depending on the thermal procedure applied. Profilometer measurements (DektakXT, Bruker Massachusetts, USA) revealed a final thickness of the PI film of about 18 µm.

In addition, two types of polyimide-additive samples were prepared by adding approximately 2 m% (based on the polymer content) of either butylated hydroxytoluene or 2,4-dibromophenol to the PAA solution. Butylated hydroxytoluene serves as an antioxidant, while 2,4-dibromophenol is used as a flame retardant in real-live polymer products. Solutions were homogenized using a vortex mixer and applied to a gold coated silicon wafer as substrate. The subsequent steps were identical with the preparation of virgin polyimide thin films.

### Instrumentation and sample measurement

FT-IR measurements were carried out using a Spectrum 65 (Perkin Elmer) IR-spectrometer in attenuated total reflection (ATR) mode. The active sampling area is a square with a diameter of about 1.0 mm when using the ATR accessory (Single reflection diamond ATR – Golden Gate from Specac). The sample was placed approximately centered on the specimen holder, and an average of 6 scans from the same position was collected per sample in the range of 550–4000 cm^−1^ with a resolution of 4 cm^−1^. Using the instrument software, a mathematical background correction was applied to all spectra directly after the measurement. Further data evaluation was done using Epina ImageLab (version 4.09, Epina GmbH, Austria).

LIBS experiments were conducted using a LIBS J200 system (Applied Spectra, Inc. Fremont, CA). An overview of the measurement parameters is given in Table 1. All samples were analyzed using three parallel line scans placed roughly in the middle area, featuring a length of 8 mm and almost covering the entire specimen width. Each line comprised of 80 shots; 20 shots each were accumulated for the continuing data evaluation, resulting in a total number of 4 spectra per line and 12 spectra per sample. Subsequent data evaluation was done using Epina ImageLab (Version 4.09, Epina GmbH, Austria).

**Table 1:** Measurement parameter for J200 LIBS instrument

Laser wavelength (nm)	266
Laser energy (mJ)	3.2
Laser spotsize (µm)	100
Laser repetition rate (Hz)	20
Laser beam geometry	Circular
Stage scan speed (mm/s)	2
Atmosphere	Argon
Spectrometer	Czerny-Turner
Detector	6-channel CCD
Gate delay (µs)	0.3
Gate width (ms)	1.05
Covered wavelength range (nm)	188–1048

## Results and discussion

### IR reference method

**Fig. 2:**
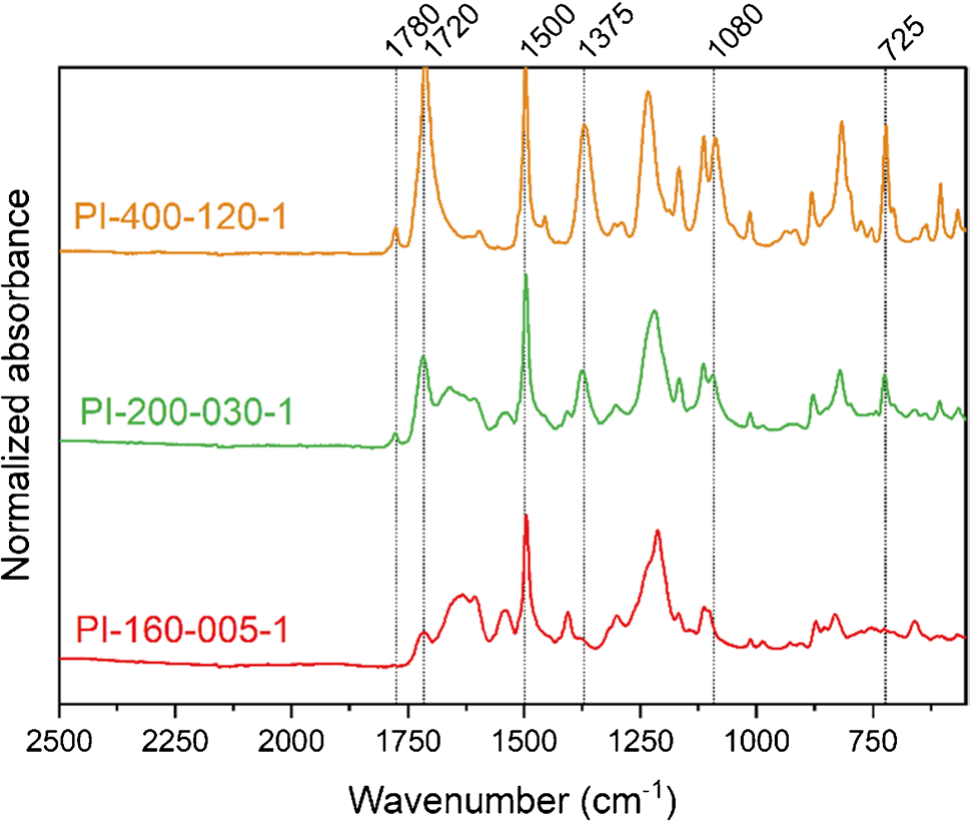
Specific wavenumber region from 550 to 2500 cm.^−1^ (average of 6 scans) of polyimides cured at 400°C for 120 min (PI-400–120-1), 200°C for 30 min (PI-200-030–1), and 160°C for 5 min (PI-160-005–1)

FT-IR spectroscopy is a state-of-the-art technique to monitor the condensation reaction of polyimides and calculate the imidization degree. IR measurements have been performed for all samples exhibiting different imidization temperatures *T*_c_ and imidization times *t*_c_. To illustrate the advancement of the imidization reaction, three representative samples including a hardly imidized film (PI-160–005-1), a semi-imidized film (PI-200–030-1), and a completely cured polyimide film (PI-400–120) are shown in Fig. 2. In order to detect the bond changes during the imidization reaction, assignments of the absorption bands are necessary [10, 15, 16]. IR spectra were recorded in the range of 550–4000 cm^−1^, but all relevant bands can be found between 550 and 2500 cm^−1^ (Fig. 2). The formation of the imide bond in the course of the reaction is confirmed by the occurrence of the C = O stretch (imide I) at 1770–1780 cm^−1^ and 1720–1740 cm^−1^, the C-N stretch (imide II) at 1360–1380 cm^−1^, and the C-H bend (imide III) and C = O bend (imide IV) in the ranges of 1070–1140 cm^−1^ and 720–740 cm^−1^. When *T*_c_ increases, a general increase of all the imide absorption peaks is observed, while the band of the aromatic ring stretching (C = C at 1500–1520 cm^−1^) remains unchanged since it is unaffected by the reaction.

Based on the magnitude of the imide absorption bands, the degree of imidization is calculated for every sample. In general, the degree of imidization of a cured polyamic acid precursor represents the percent conversion of the amic acid group into imide in the course of the imidization reaction. Most commonly used for the calculation of the imidization degree is the band at 1375 cm^−1^ (C-N stretching). Also, the aromatic band at 1500 cm^−1^ is chosen as a reference. To determine the degree of imidization, the ratio of the area of the imide band at 1375 cm^−1^ to the area of the C = C stretch of the aromatic cycle at 1500 cm^−1^ has to be referenced to the ratio of a thoroughly cured film [10, 15–17]. In this case, the sample imidized at a curing temperature of 400°C for 120 min was used as a maximum cured sample. The equation for calculating the percent degree of imidization is

1 $$\mathrm{Imidization\, \;degree}\;(\%)= \frac{{}^{{A}_{1375}}\!\left/ \!{}_{{A}_{1500}}\right.}{{}^{{A{'}}_{1375}}\!\left/ \!{}_{{A{'}}_{1500}}\right.}\times100$$

where *A* is the peak area of the measurement sample and *A*’ is the peak area the maximum imidized film at a curing temperature of 400°C for 120 min.

**Fig. 3:**
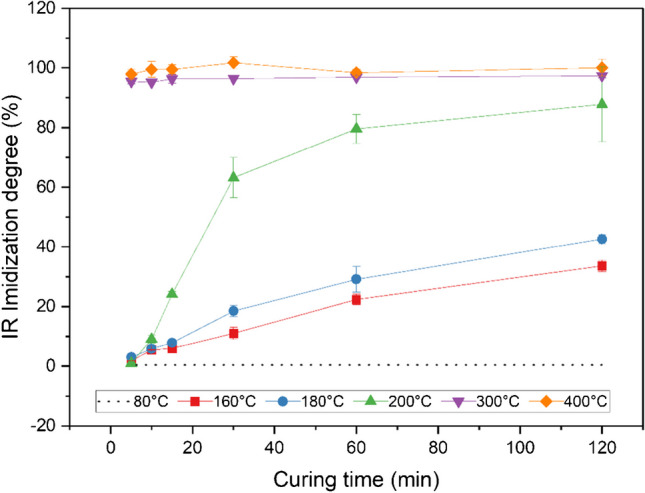
Degree of imidization for self-synthesized polyimides subjected to varying curing temperatures and times based on FT-IR spectroscopy (average of 4 samples per exposure, 6 scans per sample)

The extent of imidization for the self-synthesized polyimide samples as a function of the curing temperature *T*_c_ and time *t*_c_ is illustrated in Fig. 3. The samples solely exhibited to solvent evaporation at 80°C for 120 min (dotted line) mark the starting point of the imidization reaction where practically no conversion from the amic acid group into the imide has occurred. For all other polymer films, different conversion rates have been achieved depending on the thermal treatment applied, whereat 4 samples per exposure were averaged for every data point. In general, higher *T*_c_ and *t*_c_ resulted in a higher degree of imidization. Curing at temperatures below 200°C, more precisely at 160°C and 180°, led to medium-imidized samples after a *t*_c_ of 120 min. At 200°C, the reaction took place more rapidly leading to conversion rates of 87 ± 13% (*n* = 4) after the same curing time. Maximum imidization was reached after curing at 300°C or 400°C, where even 5 min of thermal treatment led to an imidization degree of 95% ± 1% and 98% ± 2% (*n* = 4), respectively, and the course of the curve remained almost constant. It should also be noted that the selection of the maximum cured film used as a reference in Eq. 1 has a considerable effect on the calculated imidization degree. In this case, the maximum imidized samples were chosen based on the temperature program (using the highest temperature *t*_c_ = 400°C and longest curing time *T*_c_ = 120 min). Since it is assumed that the imidization at the reference samples reaches 100% although the real conversion is not known, this can result in calculated imidization degrees > 100% (refer to PI-400–30 exhibiting an imidization degree of 102% ± 2% (*n* = 4)).

### LIBS method development

**Fig. 4:**
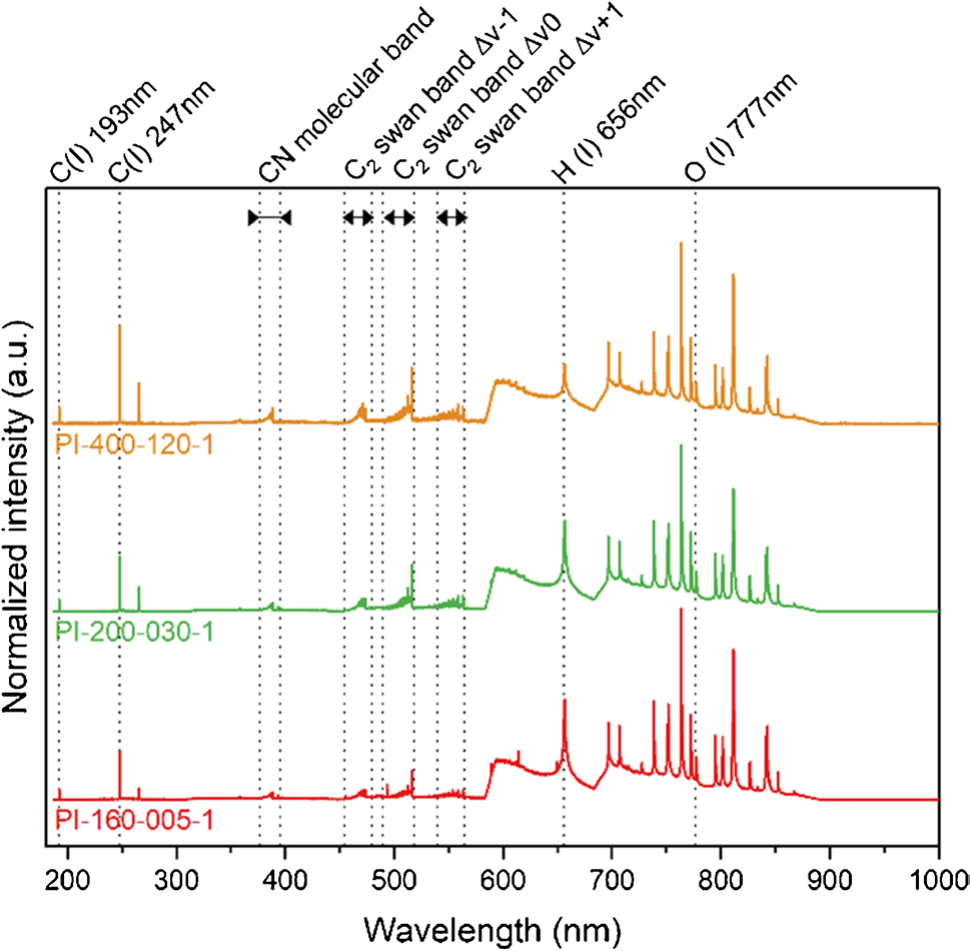
Representative LIBS spectra (average of 20 spectra, normalized to constant sum of squares) of the investigated polyimides showing different degrees of imidization. Polymer-specific emission bands with varying intensities and ratios are observed

To establish a measurement protocol, exactly the same 130 samples of self-synthesized PMDA-ODA, which were previously investigated by FT-IR, were analyzed using LIBS. Figure 4 shows representative LIBS spectra of the investigated polyimide thin films, exhibiting different amide acid to imide conversion rates. All of the displayed spectra correspond to an average of 20 shots and were normalized to constant sum of squares to compensate for shot-to-shot variations. Emission signals including atomic and molecular emission bands from major polymeric components were assigned, and varying intensities in the course of the imidization reaction were observed. While a decrease in the H(I) emission line at 656 nm was prominent when comparing a hardly imidized sample cured at 160 °C for 5 min (PI-160–005-1) with a fully imidized polymer film submitted to 400 °C for 120 min (PI-400–120-1), the C_2_ emission bands in the wavelength range from 450 to 570 nm as well as the C(I) emission line at 247 nm showed increasing signal intensity with the advancing imidization reaction.

**Fig. 5:**
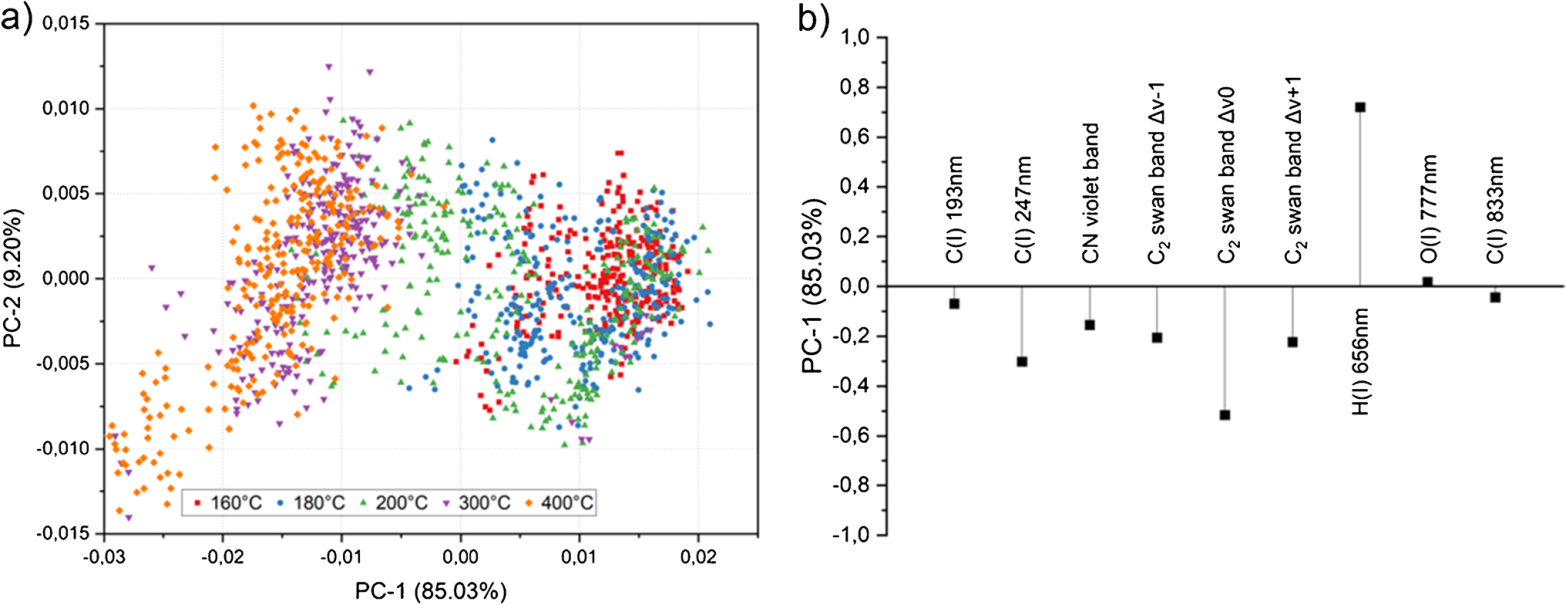
Score plot (**a**) of PC1 vs PC2 and loading plot (**b**) of PC1. The dataset was colored based on the curing temperature *T*_c_

To get an overview of the data structure and the effects of each signal, the dataset consisting of all averaged (*n* = 20) and normalized spectra from a total of 130 samples and 12 spectra per sample, representing imidization degrees from the lowest to the largest amount possible, was subjected to a principal component analysis (PCA). Background-corrected and integrated emission signals from all major polymeric components were used as variables (as marked in Fig. 4) and were mean-centered before calculating the PCA. The variance explained by each principal component (PC) was 85.03% for PC1, 9.20% for PC2, 4.06% for PC3, and < 1% for the remaining PC. The score plot of PC1 vs. PC2 in Fig. 5a reveals that the data points are fanned out along the *x*-axis (PC1), according to the curing temperature *T*_c_ which is reflected in the color coding of the data set. This corresponds well with the high amount of variance explained by PC1 (85.03%). The loading plot in Fig. 5b illustrates the influence of each input variable on the first principal component. The hydrogen signal is positively correlated to all other variables and showed a high influence in shifting PC1 into a positive direction, whereas the variables related to the C_2_ swan band Δv 0 had the most negative effect on PC1.

**Fig. 6:**
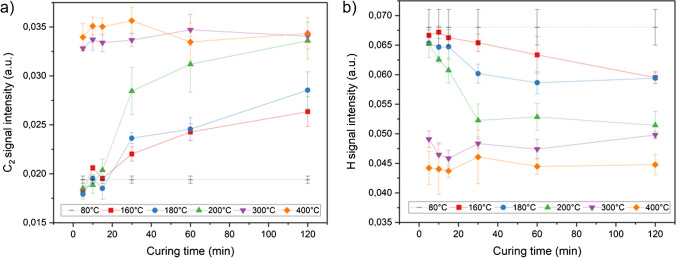
LIBS signal trends (*n* = 4) for hydrogen and the C_2_ swan band based on *T*_c_ and *t*_c_

Based on the results of the PCA, the LIBS signal trends for the hydrogen emission as well as the C_2_ swan band were further investigated. The mean signal intensity and standard deviation for each curing temperature *T*_c_ and curing time *t*_c_ were calculated by averaging the 12 accumulated and normalized spectra per sample first, and using this mean value as an initial value for this specimen, resulting in 4 values per exposure condition (analogous to the IR measurements). The signal course for each curing temperature *T*_c_ as a function of the curing time *t*_c_ is depicted in Fig. 6 where a clear trend is visible. Over the curing period *t*_c_, the signal intensity of the C_2_ emission band increases, while the hydrogen signal decreases. Processing from a polyimide film from a PAA precursor at temperatures > 150°C involves imidization with dehydration and the evaporation of residual solvent. Consequently, the loss of hydrogen signal intensity in the course of the imidization reaction could be attributed to the evaporation of water and solvent, while the growth of the C_2_ signal intensity might be due to structural changes within the polymer [24, 26, 28], e.g., the formation of the imide bond and increasing chain rigidity in this case.

As described in the previous chapter, a quantitative measure for the imidization degree can be calculated when using IR spectroscopy. This indicator is especially useful, when estimating the progress of the imidization reaction or to compare different sets of samples in a real-life application. Therefore, the aim was to introduce a similar figure for the proposed LIBS method. As the hydrogen emission and the C_2_ swan band show a trend during the imidization reaction whereat the hydrogen signal gradually decreases while the C_2_ signal steadily increases, both signals are basically suitable for monitoring the reaction progress. In order to resolve the reaction trend as accurately as possible, the ratio of the C_2_ intensity to the H emission signal was introduced. Analogous to the formula of the imidization degree based on IR spectroscopy, reference samples are also necessary for the calculation using LIBS emission signals. Because the two LIBS bands (C_2_ and H) occur in non-imidized samples as well, the stated formula based on IR spectroscopy (Eq. 1) had to be adapted. Therefore, a non-cured as well as a maximum cured polyimide sample is used for scaling, as can be seen in Eq. 2:

2 $$\mathrm{Imidization\,\;degree\,}\;(\%)= \frac{{}^{{C}_{2}}\!\left/ \!{}_{H}\right.- {}^{{C}_{2,min}}\!\left/ \!{~}_{{H}_{min}}\right.}{{}^{{C}_{2,max}}\!\left/ \!{}_{{H}_{max}}\right.- {}^{{C}_{2,min}}\!\left/ \!{}_{{H}_{min}}\right.}\times100$$

**Fig. 7:**
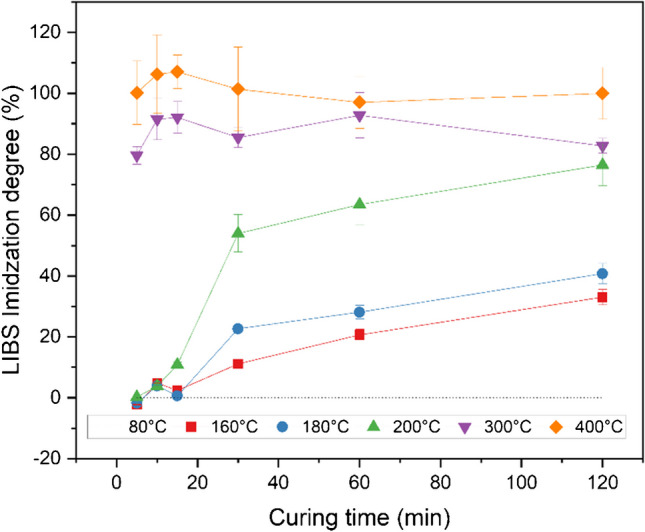
Degree of imidization for self-synthesized polyimides subjected to varying curing temperatures and times based on LIBS measurements (*n* = 4)

Equation 2 was established to calculate the imidization degree based on the C_2_ and H signal variations during the imidization reaction, where C_2_ and H represent the background-corrected, integrated LIBS emission intensities for the C_2_ swan band and the H atomic line of the sample. C_2,min_ and H_min_ depict the same LIBS signals for a merely dried, not cured polyimide sample in contrast to C_2,max_ and H_max_, which derive from the respective emission intensities of a maximum cured polymer sample. Figure 7 displays the corresponding LIBS imidization degrees for the fabricated model samples as a function of the curing temperature *T*_c_ and curing time *t*_c_. C_2,min_ and H_min_ were obtained from the average of all samples solely exhibited to the solvent evaporation at 80°C (PAA-080–120-1 to 10, *n* = 10), whereas C_2,max_ and H_max_ represent the average of all specimen cured at 400°C for 120 min (from PI-400–120-1 to 4, *n* = 4). According to Fig. 7, the imidization degree increases with *T*_c_ and *t*_c_ for all samples cured at temperatures from 160 to 200°C. For temperatures 160°C and 180°C, a medium-imidized polymer film was obtained after *t*_c_ of 120 min (33 ± 7.5% at 160°C, 41 ± 8.1% at 180°C, *n* = 4), while curing at 200°C led to a higher imidized sample (69 ± 9.9%, *n* = 4) due to enhanced reaction rates. Exposure at 300°C or 400°C led to nearly constant conversion rates, while the maximum amid acid to imide conversion was obtained while curing at 400°C with imidization degrees from 97 to 107% and RSD from 5.1 to 13.6% (average for all samples cured at 400°C and various curing times, *n* = 24).

### Comparison of IR- and LIBS-based imidization degrees

**Fig. 8:**
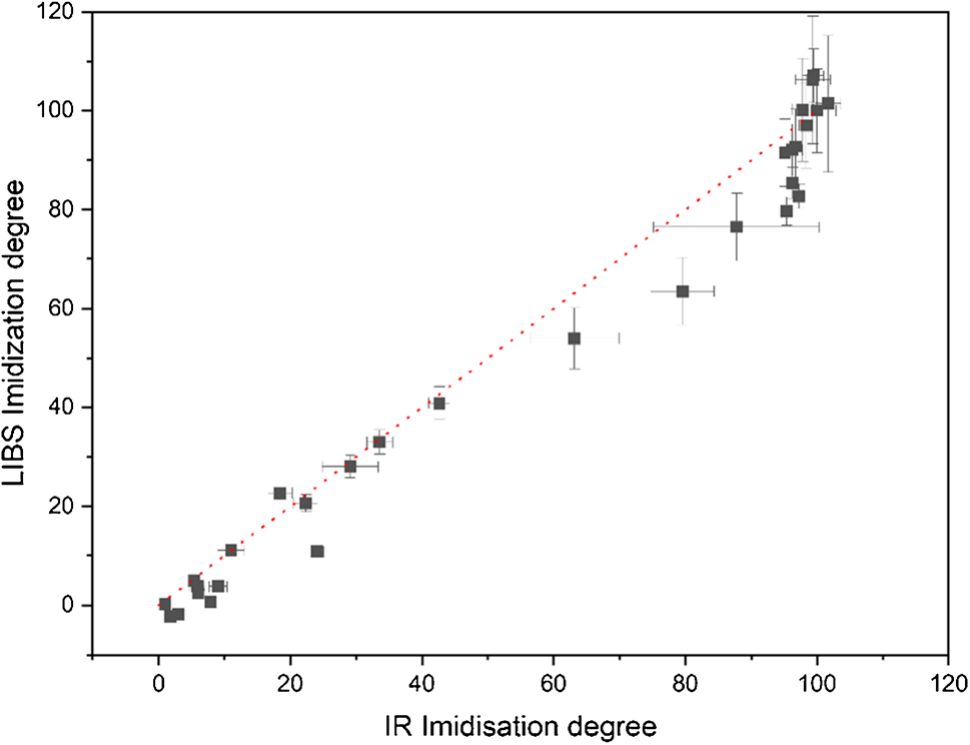
Comparison of IR- and LIBS-based imidization degrees

The previous chapter shows that the monitoring of the imidization reaction of polyimides using LIBS is possible. Especially when considering the mapping and depth profiling abilities, LIBS could become a vital tool to ensure constant material properties. One highly interesting application area could be the investigation of films and coatings because they require homogeneous attributes throughout the entire thickness. As the calculation of a quantitative measure like the imidization degree is extremely helpful for industry applications, a comparison of calculated imidization degrees based on IR spectroscopy as well as LIBS measurements is displayed in Fig. 8. With the red line marking exactly the same results for both methods, it is shown that both techniques yield very similar outputs up to an imidization degree of 50%. Beyond that, LIBS tends to provide lower results compared to IR spectroscopy, but the course of the trend is depicted for both measurement protocols. Considering the error, IR has a relative standard deviation of 6% on average for all polymer films and imidization degrees, with the error decreasing at higher imidization degrees (8% at 160 °C, 2% at 400 °C). In contrast, the average relative standard deviation for LIBS is 8% across all samples and curing conditions. However, the error increases with increasing imidization (5% at 160 °C, 10% at 400 °C). Differences in imidization degree and precision can be expected due to the different cross-sections of the two methods. First of all, the ATR-IR penetration depth is approximately 1.3 µm at 1500 cm^−1^ and changes with wavenumber, while the LIBS ablation depth is around 1 µm. In addition, the active sampling area for ATR-IR spectroscopy is about 0.5 mm^2^ representing the size of the ATR crystal of the used instrument, and all 6 scans were measured and averaged from that same spot. Thus, the outcome reflects only deviations resulting from sample measurement and between samples but not variations in sample homogeneity. For LIBS, on the contrary, an area of 2.4 mm^2^ was sampled using 3 parallel line scans, meaning that the sample was scanned along its entire width and variations in reaction rate due to sample preparation were included in the signal.

Furthermore, the two analytical methods differ not only in relation to the sampling volume but also in the information they collect. IR spectroscopy detects characteristic vibrations of functional groups within the molecule. The calculation of the degree of imidization relies on the ratio of two vibrational bands, facilitating a direct observation of the bond formation during the imidization reaction, especially through the utilization of the imide band in the underlying formula (see Eq. 1). In contrast, LIBS is a method for elemental analysis and does not provide information about the molecular structure or the formation of certain bonds directly. Instead, the determination of the imidization degree is based on the ratio of emission signals, more precisely the C_2_ and H intensities (see Eq. 2). Both signal trends correlate with the progress of the imidization, probably due to the evaporation of water and solvent and the structural changes associated with the reaction.

All of this must be taken into account when comparing imidization degrees obtained from different analytical techniques. Nevertheless, a relative comparison within a sample set using either IR or a LIBS based imidization degree is possible.

### Depth profiling

**Fig. 9:**
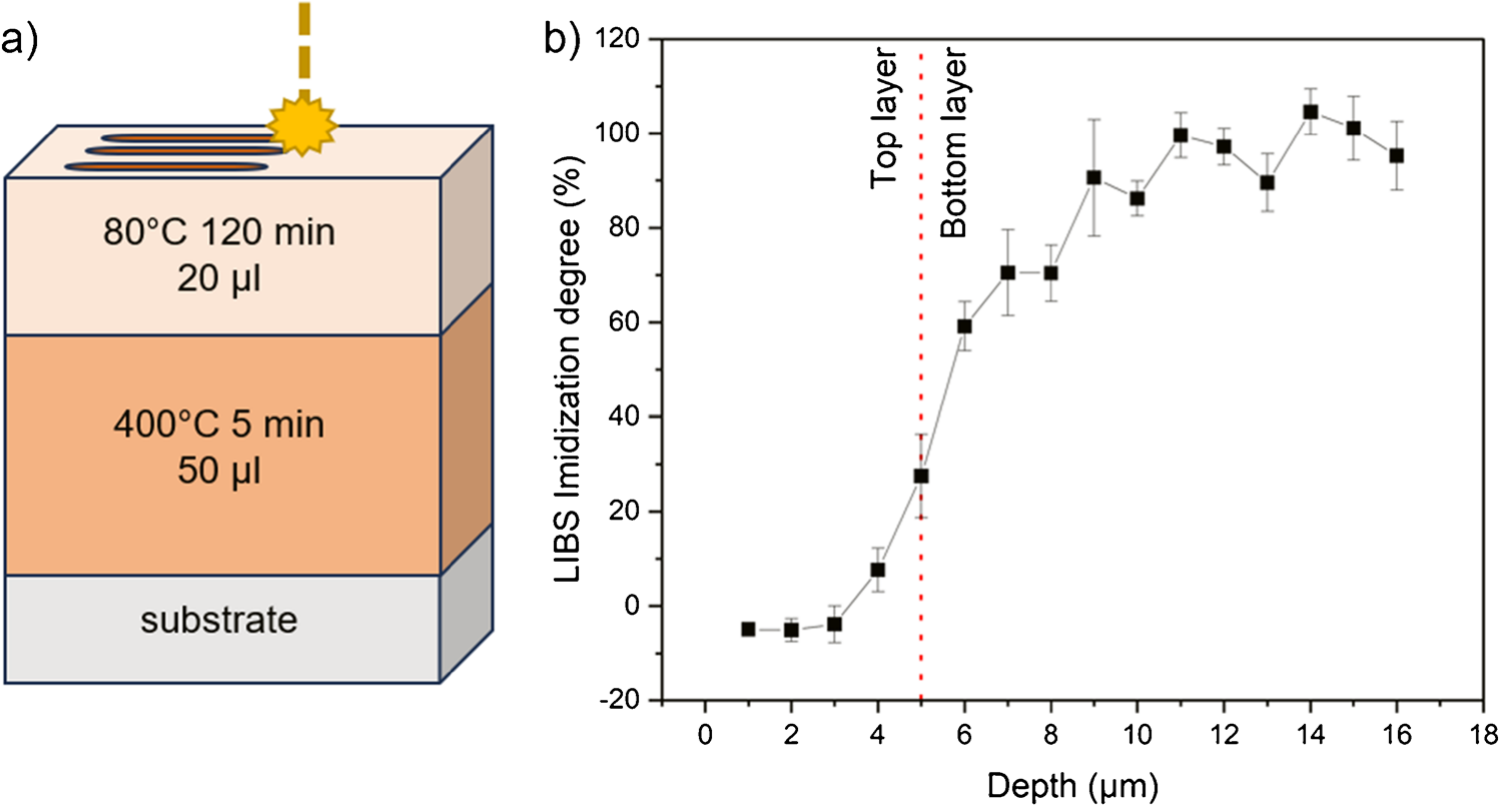
Schematic of the model sample prepared for depth profiling (**a**) and LIBS imidization degree showing the increase of the imidization degree with depth (**b**)

Conventional techniques used for the investigation of the imidization reaction like DSC, TGA, or FT-IR are mainly used for bulk analysis, and lateral and depth-resolved information is lost. One huge advantage of LIBS is its depth profiling capability. This could be especially useful, when a layer construction is under observation or if the defective area is not at the sample surface during failure analysis. To assess the applicability of the aforementioned LIBS method for layered structures, a stacked model sample was prepared from PAA solution. A first layer of precursor (50 µl) was thermally treated at 400°C for 5 min. Subsequently another layer of precursor (20 µl) was added on top and dried at 80°C for 120 min, according to Fig. 9a. Depth profiling was done starting from the top layer dried at 80°C down to the bottom layer cured at 400°C. A total number of 16 layers, each representing 1 µm in ablation depth, were ablated, and the average of every layer was calculated for data evaluation. The same samples used for the LIBS method development served as reference to calculate the imidization degree: PAA-080–120-1 to 10 were used to obtain C_2,min_ and H_min_, while PI-400–120-1 to 4 were analyzed for C_2,max_ and H_max_ according to Eq. 2. Figure 9b shows the LIBS imidization degree for the layered samples, indicating an increase of the imidization degree after 4 µm. The trend of the depth profile and the calculated imidization degree rising from 0 to 100% corresponds well with the sample structure, as we can assume that the top layer was hardly or not imidized at all, while the bottom layer was completely imidized. Additionally, it has to be kept in mind that the course of the curve in the transition region is dependent on intermixing or solving effects when applying the top layer in the form of the PAA precursor dissolved in NMP as well as the generated crater shapes derived from a Gaussian laser beam profile.

### Investigation of industry samples

In a real-life scenario, it is very unlikely to encounter a sample consisting only of a pure polyimide precursor. To ensure the robustness of the method, two common aromatic additives found in commercial polymer products were added to the self-synthesized precursor solution and analyzed after thermal treatment. To push things further, two commercially available and industry-relevant polyimide films were investigated using IR spectroscopy as well as LIBS. In both cases, the imidization degree was calculated using self-synthesized polyimide films as reference points.

#### Addition of additives

Butylated hydroxytoluene which serves as an antioxidant as well as 2,4-dibromophenol functioning as a flame retardant in commercial polymers was added to the self-synthesized PAA solution. Polyimide films with different conversion ratios of the amic acid vs. imide group, respectively, imidization degree were fabricated via thermal imidization either at 200° for 30 min or 400°C for 60 min. The calculation of the imidization degree was possible for both methods: according to Eq. 1, the pristine film cured at 400°C for 120 min (PI-400–120-1 to 4) served as a fully cured sample for IR spectroscopy, while for the LIBS method, non-cured (PAA-80–120-1 to 10) as well as maximum cured polyimides (PI-400–120-1 to 4) were used based on Eq. 2.

For the additive-doped samples cured at 400°C for 60 min, imidization degrees of 103 ± 2% (for butylated hydroxytoluene addition, *n* = 4) and 103 ± 3% (for 2,4-dibromophenol addition, *n* = 4) were obtained with IR, while LIBS measurements resulted in an imidization degree of 107 ± 7% (*n* = 4) and 108 ± 4% (*n* = 4) for the same samples. Thermal treatment at 200°C for 30 min resulted in medium-imidized pristine and additive-doped samples. Using IR spectroscopy, imidization degrees of 50 ± 1% (for pristine polyimide, *n* = 4), 50 ± 6% (for butylated hydroxytoluene addition, *n* = 4), and 43 ± 3% (for 2,4-dibromophenol addition, *n* = 4) were received. Analyzing the same samples with LIBS resulted in the following imidization degrees: 44 ± 3% (for pristine polyimide, *n* = 4), 38 ± 2% (for butylated hydroxytoluene addition, *n* = 4), and 38 ± 2% (for 2,4-dibromophenol addition, *n* = 4). These results show that the calculations and results within one technique are consistent, and no influence of additives on the performance of the established IR as well as the proposed LIBS method was detected although the results of each analysis method differ ((IR, 50 ± 0.6%, and LIBS, 44 ± 3% for the pristine sample cured at 200°C for 30 min). This effect was already apparent when comparing IR and LIBS results during method development and can be expected since different cross-sections regarding penetration/ablation depth and sampling areas are taken into account using the two techniques. Also, the selection of appropriate standards used as a reference for minimum and maximum imidized samples is crucial, since they serve as a reference point for calculating the degree of imidization and choosing a different set of reference standards might shift the calculated values accordingly.

#### Commercial polyimides

**Fig. 10:**
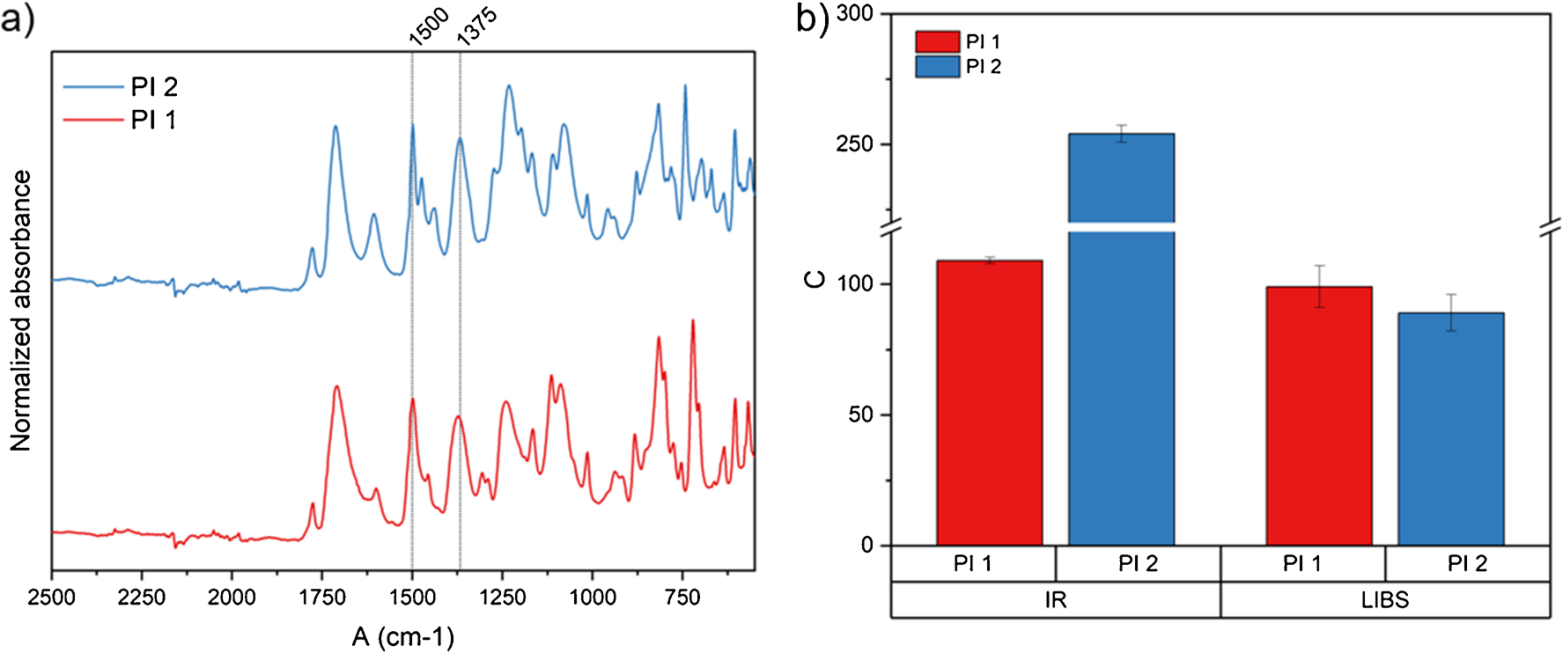
FT-IR spectra of two commercially available polyimide films (**a**) and a comparison of IR- and LIBS-based imidization degrees based on self-synthesized polymer films (**b**). The IR spectra of PI2 differ from the self-synthesized reference leading to unnaturally high IR imidization degrees

Since the addition of common aromatic additives to the precursor solution did not prevent the determination of the imidization degree of the cured polyimide films, the applicability of the LIBS method was tested using two commercially available products. Self-synthesized polyimides were used as reference material according to Eqs. 1 and 2 to ensure the versatility of the implemented method. This could be especially useful in cases where there is no matching raw material available for example during failure analysis. The two commercial polyimide films used for testing were a general-purpose polyimide film (PI1) used for applications in fiber optics cables, insulation tubings, or as diaphragm sensors and manifolds in the automotive industry and another polyimide (PI2) based on a photosensitive precursor common in the electronics industry [29]. Both films were first analyzed using IR spectroscopy. The ATR-IR spectra of both samples and the bands used for determining the imidization degree are displayed in Fig. 10a. It can be seen that the spectra of both polyimides differ; especially for PI2, this is troublesome as the band of the aromatic ring stretching (C = C) around 1500 cm^−1^ is normally used as reference and the determination of the peak area is affected. For that reason, determining the imidization degree using the traditional IR approach works for PI1 but leads to an unnaturally high imidization degree for PI2, as can be seen in Fig. 10b. Figure 10b also shows, that using LIBS, both samples were accessible for analysis, and the determination of the imidization degree using self-synthesized reference films was applicable.

## Conclusion

This work demonstrates the applicability of LIBS for the monitoring of the imidization reaction of polyimides. It was possible to track the reaction using the C_2_ emission band as well as the H signal. IR spectroscopy served as a reference during method development and provided a template to implement a simple formula to calculate the imidization degree based on the LIBS signal trends of C_2_ and H. A non-cured as well as a maximum cured polyimide sample had to be used for scaling since the two LIBS bands also occur in non-imidized samples as well. Using the novel formula, the calculation of imidization degrees obtained using LIBS was possible, and the outcome was in good accordance with IR results. Observed differences could be attributed to different cross-sections of the two methods, regarding penetration depth and sampling area. Using LIBS, an area almost 5 times larger than with IR spectroscopy was analyzed, which meant that local variations due to sample preparation and reaction rate were included in the signal. In addition, the calculation of the imidization degree is based on the ratio of two vibrational lines using IR spectroscopy, providing information about changes in the molecular structure, while the formula based on LIBS uses the ratio of C_2_ and H emission signals, indicating alterations in the elemental composition. Therefore, degrees of imidization from two different analysis methods cannot provide exactly the same numerical value, but a relative comparison within a sample set using IR or LIBS based results is possible. Nevertheless, method improvements using additional spectral information in the form of elemental or molecular bands, the selection of alternative standards as reference values, etc. would be possible. In this work, the simplest possible formula was chosen deliberately to make data evaluation and the calculation of the imidization degree as practicable and easy as possible.

To test the applicability and versatility of the LIBS method, depth profiling of a stacked model sample prepared from PAA solution was conducted. The trend of the depth profile and the calculated imidization degree corresponded well with the sample structure, and no influence of the laser-mater interaction on the subsequent layers could be detected. Furthermore, additive-doped self-synthesized films and commercially available polyimide films were investigated, and in both cases, the determination of the imidization degree using LIBS was feasible. Especially in the case of the commercial samples, where polyimide structure was unknown, LIBS was able to provide a more universal approach than IR spectroscopy. These results indicate the potential of LIBS as a viable tool in quality assurance or failure analysis of polyimide samples in a real-life scenario, especially when considering its depth profiling and in-line monitoring capabilities.

## Data Availability

Data will be made available from the authors on reasonable request.
